# A53-Adapted Decitabine-Containing R-CHOP in Newly Diagnosed Diffuse Large B-Cell Lymphoma Defined by LymphGen: Preliminary Results of a Prospective Proof-of-Concept Study

**DOI:** 10.3390/jcm15155844

**Published:** 2026-07-27

**Authors:** Marat Mingalimov, Elena Baryakh, Polina Chernova, Andrey Misyurin, Elena Misyurina, Mariia Orlova, Tatiana Tolstykh, Ekaterina Zotina, Liliia Shimanovskaia, Tatiana Chudnova, Olga Kochneva, Kseniya Tsurkina, Dmitry Lebedev, Georgii Tyshkevich, Viktoriia Basova, Mira Suvorina, Mikhail Donskoy, Ivan Abramov, Natalia Bodunova, Saida Gadzhieva, Tatiana Semina, Sergey Andreev, Mariana Lysenko

**Affiliations:** 1 Moscow Clinical Science and Research Center 52, Moscow 109428, Russia; baryakh_e_a@staff.sechenov.ru (E.B.); misyurina_e_n@staff.sechenov.ru (E.M.); tolstykh_t_n@staff.sechenov.ru (T.T.); zotina_e_n@staff.sechenov.ru (E.Z.); vagizova2016@list.ru (L.S.); chudnova_t_s@staff.sechenov.ru (T.C.); kochneva_o_l@staff.sechenov.ru (O.K.); tsurkina_k@zdrav.mos.ru (K.T.); korsar666666@gmail.com (D.L.); andreevss@mos.ru (S.A.); lysenkoma@mos.ru (M.L.); 2 Department of Hematology, I.M. Sechenov First Moscow State Medical University—Ministry of Health of Russia (Sechenov University), Moscow 119991, Russia; chernova_p_s@student.sechenov.ru (P.C.); orlova_m_s@student.sechenov.ru (M.O.); 3 N.I. Vavilov Institute of General Genetics, Moscow 119991, Russia; vvvbasovavvv@gmail.com (V.B.); iogen@vigg.ru (M.S.); 4 FSBEI FPE «Russian Medical Academy of Continuous Professional Education» of the Ministry of Healthcare of the Russian Federation, Moscow 125993, Russia; and@genetechnology.ru; 5 FSBI FPE «Central State Medical Academy» of the Administrative Department of the President of the Russian Federation, Moscow 121359, Russia; info@cgma.su; 6 JSC «European Medical Center», Moscow 129110, Russia; donskoyma@yandex.ru; 7 SBIH Moscow Clinical Scientific and Practical Center Named after A.S. Loginov of DHM Moscow, Moscow 111123, Russia; abriv@bk.ru (I.A.); bodunovana@zdrav.mos.ru (N.B.); 8 Moscow Healthcare Department, 43 Oruzheynyy Pereulok, Moscow 127006, Russia; gadzhievasm@mos.ru (S.G.); seminata@mos.ru (T.S.)

**Keywords:** diffuse large B-cell lymphoma, TP53, LymphGen, A53 subtype, decitabine, epigenetic therapy

## Abstract

**Background**: Diffuse large B-cell lymphoma (DLBCL) with TP53 abnormalities, corresponding to the LymphGen A53 molecular subtype, represents a biologically high-risk group associated with primary resistance to standard R-CHOP therapy. Epigenetic sensitization using hypomethylating agents may enhance chemosensitivity in this setting. We prospectively evaluated the clinical activity and safety of a molecularly adapted DAC-R-CHOP regimen in newly diagnosed A53-DLBCL. **Methods**: In this single-center prospective pilot cohort study, 70 consecutive patients with newly diagnosed DLBCL underwent targeted next-generation sequencing using a 60-gene panel with integrated copy number variation analysis. Six patients (8.5%) were classified as the A53 subtype. All patients received one cycle of standard R-CHOP. From cycle 2 onward, A53 patients received decitabine (10 mg/m^2^ IV, days 1–5) prior to R-CHOP (DAC-R-CHOP), for a total of six cycles. The primary endpoint was complete metabolic response (CMR) according to Lugano 2014 criteria. Exact 95% confidence intervals (CI) were calculated. **Results**: The median age of the A53 cohort was 65 years. CMR was achieved in all six patients (100%; 95% CI, 54–100%). At a median follow-up of 6 months, all patients remained alive in confirmed CMR. Grade III–IV hematologic toxicity occurred in all cases. Febrile neutropenia developed in 100% of patients, requiring mandatory G-CSF support and anti-infective therapy; no treatment-related mortality or permanent dose reductions were observed. Two patients (33%) experienced gastrointestinal bleeding related to local tumor lysis, which was managed conservatively without protocol discontinuation. **Conclusions**: In this prospective molecularly stratified pilot cohort, integration of decitabine into front-line immunochemotherapy showed promising clinical activity in A53-DLBCL, albeit at the cost of substantial hematologic toxicity requiring intensive supportive care. Given the small sample size, short follow-up, and absence of a comparator arm, these findings should be considered hypothesis-generating and warrant validation in larger multicenter phase II studies with integrated translational biomarker analyses.

## 1. Introduction

Diffuse large B cell lymphoma (DLBCL) is a heterogeneous group of tumors characterized by broad clinical, morphological, immunophenotypic, and genetic diversity [1]. Modern molecular genetic classifications have enabled stratification of DLBCL into biologically discrete subtypes with differential sensitivity to therapy [2]. Of particular clinical relevance is the A53 subtype as defined by the LymphGen algorithm, whose key molecular hallmarks include inactivation of the TP53 gene (resulting from point mutations or deletions) along with associated genomic instability or aneuploidy [3].

The role of TP53 aberrations as a universal driver of tumor progression and therapeutic resistance is being actively investigated across various B cell neoplasms. Among lymphoproliferative disorders, the prognostic and predictive significance of these alterations has been most thoroughly characterized in chronic lymphocytic leukemia and mantle cell lymphoma. In these entities, TP53 deletions and mutations are equally powerful adverse prognostic factors and are tightly linked to primary resistance to immunochemotherapy [4,5]. The molecular landscape of TP53 is remarkably heterogeneous: aberrations may manifest as biallelic disruptions, isolated del(17p), or point mutations, occurring in approximately 60%, 10%, and 30% of cases, respectively [6]. In the current era of targeted agents, the clinical value of identifying TP53 abnormalities has expanded beyond pure prognostication—it has become a predictive biomarker that guides the selection of optimal therapeutic strategies [7]. These observations have provided the rationale for an in depth exploration of TP53 aberrations in other aggressive B cell lymphomas. In DLBCL, loss of TP53 function similarly drives primary resistance to standard R CHOP immunochemotherapy, defining a patient subset with an extremely poor prognosis [8,9].

The prognostic impact of TP53 aberrations in DLBCL has been convincingly validated in both retrospective and prospective studies [10,11]. Large cohort studies employing high throughput sequencing have identified TP53 mutations in 20–25% of patients, establishing them as an independent predictor of significantly inferior overall and event free survival [3,11]. Clinically, this failure stems from the inability of conventional regimens to overcome the chemoresistance conferred by TP53 gene aberrations [12]. The key molecular underpinnings of this phenomenon include abrogation of cell cycle checkpoint control, profound disruption of programmed cell death (apoptosis) pathways, and an impaired cellular response to the DNA damaging effects of cytotoxic agents [13]. Collectively, these alterations enable the tumor clone to maintain a high proliferative drive even under the intense cytotoxic stress induced by therapy.

Given the biological features of TP53-aberrant DLBCL outlined above, integration of epigenetic agents into first-line therapy represents a rational strategy to overcome resistance, a concept supported by both preclinical models and early-phase clinical studies in related disease settings [14,15,16]. Decitabine, a DNA methyltransferase inhibitor, can reactivate epigenetically silenced tumor suppressor genes, apoptosis regulators, and major histocompatibility complex molecules, thereby resensitizing malignant cells to cytotoxic insult [17]. An additional, immunologically relevant mechanism of decitabine involves the induction of viral mimicry. Demethylation of endogenous retroviral sequences triggers transcription of double stranded RNA, activates interferon-dependent innate immune responses, and may substantially enhance rituximab-mediated antibody-dependent cellular cytotoxicity [14].

Despite the compelling and consistent biological and preclinical rationale, prospective clinical data on the efficacy and safety of hypomethylating agents combined with R CHOP in patients with TP53-aberrant DLBCL—particularly those with the confirmed A53 molecular subtype—remain extremely scarce. In this context, the present pilot, single center study was designed to assess the complete metabolic response rate and toxicity profile of the molecularly adapted DAC R CHOP regimen in patients with newly diagnosed DLBCL and a verified A53 subtype.

## 2. Materials and Methods

### 2.1. Study Design and Patients

This report presents a prespecified interim analysis of a single-center, proof-of-concept pilot protocol in patients with molecularly defined DLBCL enriched for the LymphGen A53 subtype. The planned enrollment target is 15 A53-enriched patients. Recruitment remains ongoing at the time of manuscript submission. The current analysis reflects a data cutoff date of 20 May 2026, and includes the first six evaluable patients.

The present study was designed as a prospective, single-center cohort analysis. All cases examined herein were originally enrolled in a prospective single-center clinical trial, the design of which was developed in full accordance with the principles of evidence-based medicine and international standards for Good Clinical Practice [18]. The study was conducted in strict accordance with the ethical principles of the Declaration of Helsinki. The protocol underwent independent ethics review and was approved by the local ethics committee of the Moscow Clinical Research Center 52 (Moscow Healthcare Department). Before any study procedures were initiated, all patients received detailed information regarding the objectives and potential risks and provided written informed consent for participation, collection of clinical and anamnestic data, and comprehensive molecular genetic profiling of tumor material.

The overall analytic cohort comprised 70 patients with newly diagnosed diffuse large B cell lymphoma. The diagnosis was confirmed by comprehensive morphological and immunohistochemical analysis according to the current criteria of the 5th edition of the World Health Organization classification of tumors of hematopoietic and lymphoid tissues [19]. Patients were enrolled prospectively and consecutively from September 2023 onward. Key eligibility criteria included: age between 18 and 80 years; Ann Arbor stage I–IV disease; Eastern Cooperative Oncology Group (ECOG) performance status 0–4. Additional considerations included clinical suitability for and feasibility of induction immunochemotherapy with the R CHOP regimen, as well as the absence of absolute contraindications to any component of the backbone R-CHOP regimen or to the targeted agents planned for incorporation into the protocol (acalabrutinib, decitabine, and lenalidomide).

### 2.2. Molecular-Genetic Profiling

Comprehensive molecular genetic profiling of tumor tissue was performed in all patients strictly prior to the initiation of any specific therapy. Genomic DNA was extracted from tumor specimens that had been fixed in 10% neutral buffered formalin and embedded in paraffin (FFPE blocks), or from fresh lymph node biopsy samples, followed by standard quality control assessment of the quantity and integrity of the isolated nucleic acid.

To detect somatic TP53 mutations and concomitant genetic aberrations, we employed targeted next-generation sequencing (NGS) using a customized 60-gene panel encompassing: ARID1A, B2M, BTG1, BTG2, CCND3, CD70, CD79B, CIITA, CREBBP, DDX3X, DTX1, DUSP2, EP300, EZH2, FAS, GNA13, IRF4, IRF8, KMT2D, MPEG1, MYD88, NOTCH1, NOTCH2, PIM1, PRDM1, SGK1, SOCS1, STAT3, STAT6, TBL1XR1, TET2, TNFAIP3, TNFRSF14, TP53, ZFP36L1, MTOR, NFKBIA, ETV6, ACTG1, OSBPL10, MYC, BCL2, BCL6, FOXO1, ATM, CD79A, PIK3CD, PTEN, CD5, CD58, CDKN2A, CDKN2B, ASXL1, KRAS, EPHB1, BCL10, PRKCB, PLCG2, CARD11, and MEF2B. Sequencing was performed at sufficient depth to enable reliable detection of low-frequency variants. The targeted sequencing workflow incorporated a dedicated bioinformatic copy-number analysis module to infer copy-number alterations (CNAs) from normalized read-depth patterns and to derive measures of copy-number complexity/aneuploidy, thereby enabling assessment of genomic instability features required for accurate implementation of the LymphGen classification framework. In addition, clinically relevant structural rearrangements were assessed using fluorescence in situ hybridization (FISH), providing orthogonal confirmation of rearrangement status where applicable. Molecular subtyping of DLBCL was performed according to the LymphGen genomic classification algorithm using an integrated spectrum of genomic aberrations (pathogenic sequence variants together with CNA-derived features), rather than TP53 status alone. During bioinformatic analysis, samples were classified as TP53-mutated when pathogenic variants were detected at a variant allele frequency (VAF) of ≥5%, a threshold selected to minimize sequencing artifacts and very-low-level events of uncertain interpretability. Importantly, assignment to the LymphGen A53 subtype was not based on TP53 status alone, but relied on the combination of TP53 alteration (s) with CNA patterns and copy-number complexity/genomic instability features consistent with an aneuploid, genomically unstable profile, in accordance with the published LymphGen classification pipeline. LymphGen subtype assignments were generated using a pre-specified, standardized workflow and were independently reviewed by a second analyst blinded to clinical outcomes; any discrepancies were resolved by consensus prior to final locking of subtype calls.

Based on the comprehensive molecular genetic findings, patients were stratified according to the LymphGen algorithm. Six patients (8.5%) were classified as the A53 molecular subtype; all of these patients carried pathogenic TP53 variants. The remaining 64 patients fell into other molecular DLBCL subtypes.

### 2.3. Treatment Protocol

The backbone therapeutic approach for the entire patient cohort was a molecularly adapted immunochemotherapy protocol designated R-CHOP-X, where “X” denotes targeted modifications based on the verified LymphGen subtype. Given the need for prompt initiation of specific antitumor therapy and the inherent technical timelines required for high throughput sequencing, all patients received the first induction cycle as standard R-CHOP without the addition of targeted agents.

Starting from cycle 2, after molecular profiling results and mutation status had been obtained, the therapeutic strategy was adapted. To overcome anticipated chemoresistance in patients with TP53 mutations (*n* = 6), the hypomethylating agent decitabine was integrated into first line therapy; this experimental regimen was designated DAC R CHOP. Decitabine was administered at a dose of 10 mg/m^2^ as intravenous infusions on days 1–5 of each 21 day cycle, immediately before administration of the backbone regimen components.

Patients without TP53 mutations (*n* = 64) received, from cycle 2 onward, therapy according to the R CHOP X protocol, with the incorporation of acalabrutinib or lenalidomide, strictly in accordance with clinical stage and verified disease subtype. The planned total duration of induction therapy was 6 cycles at 21 day intervals (1 cycle of standard R CHOP followed by 5 cycles of molecularly adapted therapy) for both groups. Dose modifications and clinically justified delays of subsequent cycles were performed as indicated, based on hematologic and non-hematologic toxicity as well as individual treatment tolerability.

Standardized supportive care comprised mandatory primary prophylaxis with granulocyte colony-stimulating factor (G-CSF) for all patients without exception, initiated from the first cycle of induction therapy; routine antibacterial and antifungal prophylaxis was not administered.

### 2.4. Response Assessment and Endpoints

Tumor response monitoring, including interim (after cycles 2 and 4) and final efficacy assessments, was performed using positron emission tomography combined with computed tomography (PET/CT) with the radiotracer ^18^F fluorodeoxyglucose. Metabolic response was interpreted according to the Lugano 2014 criteria, with mandatory application of the visual 5 point Deauville scale (DS). Complete metabolic response (CMR) was defined as a Deauville score of 1 to 3.

The primary endpoint of the study was the CMR rate after completion of protocol therapy in the cohort of patients with TP53 mutations who received the DAC R CHOP regimen. Secondary endpoints included overall response rate (ORR, comprising complete and partial metabolic responses), as well as detailed evaluation of the safety profile and incidence of adverse events (AEs). Adverse events were recorded and graded according to the National Cancer Institute Common Terminology Criteria for Adverse Events (NCI CTCAE), version 5.0.

### 2.5. Statistical Analysis

Given the pilot, proof-of-concept nature of this study and the limited size of the molecularly defined target population (*n* = 6), the statistical analysis was intentionally descriptive. No formal sample size calculation was undertaken because the study was not designed to test a prespecified hypothesis, and no inferential framework with a defined type I error rate was planned. Instead, the feasible sample size was determined by the expected prevalence of the LymphGen A53 subtype in unselected DLBCL (approximately 5–10% as reported in published LymphGen-based cohorts) and by practical single-center recruitment feasibility within the planned enrollment period. Continuous variables are summarized as medians with interquartile ranges, and categorical variables as frequencies with percentages.

To quantify uncertainty around response estimates in this small cohort, confidence intervals (CIs) for the CMR rate were calculated using the exact Clopper–Pearson method for binomial proportions, which is appropriate for very small samples and provides conservative coverage. Accordingly, results are presented as point estimates with exact 95% CIs, without formal comparative testing or hypothesis testing, consistent with the exploratory objectives of the study. All statistical analyses were performed in R version 4.2.2 (R Foundation for Statistical Computing, Vienna, Austria), and exact binomial confidence intervals were computed using the exactci package (function binom.exact).

## 3. Results

### 3.1. Clinical and Molecular Characteristics of Patients and Treatment Efficacy

From September 2023 onward, 70 patients with newly diagnosed DLBCL were consecutively enrolled in the study and underwent comprehensive molecular genetic profiling of tumor tissue prior to the initiation of specific therapy. Based on NGS analysis and subsequent classification according to the LymphGen algorithm, six patients (8.5%) were assigned to the A53 molecular subtype. The group comprised 3 men and 3 women; the median age was 65 years (IQR, 56–67). Advanced stage disease (III–IV by Ann Arbor) was diagnosed in 4 of 6 patients (67%). Four patients (67%) were classified as high risk according to the International Prognostic Index (IPI ≥ 3).

All identified TP53 variants were classified as pathogenic. Mutations were localized to exons 4, 6, and 7, affecting the DNA binding domain of the p53 protein. The median variant allele frequency (VAF) was 23.5% (IQR, 8–40%; range, 5–57%). The p.Arg248Trp mutation (c.742C > T, exon 7) was independently identified in two patients (patients 4 and 6). At the time of this interim analysis (data cutoff: 20 May 2026), five of six evaluable patients had completed the planned six cycles of therapy; the sixth patient remained on protocol treatment and was included in the analysis based on interim response assessment. No premature discontinuations due to toxicity or progressive disease occurred in this cohort.

A complete metabolic response was achieved in all 6 evaluable patients (100%; 95% CI, 54–100%).

Individual clinical and molecular characteristics and treatment responses of patients with TP53 mutations are presented in Table 1.

### 3.2. Toxicity Profile

The spectrum and frequency of grade III–IV adverse events are summarized in Table 2. Grade III–IV hematologic toxicity (anemia, neutropenia, and thrombocytopenia) occurred in all patients (100%). Febrile neutropenia occurred in all treated patients (6/6) and is therefore a clinically meaningful toxicity signal. Notably, G-CSF was administered as primary prophylaxis; nevertheless, febrile neutropenia still developed, underscoring the intensity of regimen-associated myelosuppression. All events were managed with inpatient care, including close clinical monitoring and intravenous broad-spectrum antimicrobial therapy, with additional supportive measures as indicated (including platelet and packed red blood cell transfusions). This supportive approach enabled treatment delivery without premature discontinuation, and therapy was administered without treatment delays in this cohort; importantly, no treatment-related deaths occurred. All febrile neutropenia episodes were observed after cycles 2 and 4, temporally coinciding with the period of decitabine integration; while causality cannot be inferred in this small cohort, this pattern highlights the need for careful prospective monitoring of hematologic toxicity during decitabine-containing cycles. In addition, two patients experienced episodes of severe gastrointestinal bleeding during induction therapy. These complications were deemed pathogenetically related to the underlying disease (specific tumor involvement of the gastric wall, tumor lysis), were successfully managed, and did not require protocol discontinuation. This further emphasizes the supportive-care burden associated with this regimen and the importance of systematic safety characterization in larger studies.

## 4. Discussion

In this prospective pilot study, the molecularly adapted DAC-R-CHOP strategy in patients with newly diagnosed DLBCL and a verified A53 subtype was associated with a high rate of complete metabolic response and a manageable toxicity profile. A CMR was achieved in all six evaluable patients, suggesting potential clinical activity of this approach in a molecularly defined population enriched for the LymphGen A53 subtype. Notwithstanding these favorable initial observations, we underscore the strictly exploratory and hypothesis-generating nature of this proof-of-concept study. Given the limited sample size (*n* = 6), the observed 100% CMR rate must be interpreted with considerable caution to avoid overstating therapeutic efficacy, and will require confirmation in larger, adequately powered trials. Despite these limitations, the finding is noteworthy because the cohort comprised patients with an inherently unfavorable molecular profile—a population historically associated with primary refractory disease and inferior outcomes with standard R-CHOP. Collectively, these results support the biological rationale for the epigenetic sensitization strategy under investigation, and the manageable toxicity profile of the regimen supports its consideration as a platform for further clinical development in this high-risk, molecularly defined setting.

TP53 gene mutations occupy a leading position in the overall landscape of genetic events in oncogenesis and are detectable in approximately half of all human malignancies. The protein encoded by this gene, p53—often referred to as the “guardian of the genome”—orchestrates the cellular response to genotoxic stress through transcriptional regulation of target genes that control cell cycle arrest, DNA repair, apoptosis, and cellular senescence [13]. Loss of p53 function resulting from point mutations, deletions, or epigenetic silencing eliminates a critical barrier to tumor progression and confers selective advantages to the tumor clone under cytotoxic stress. Nevertheless, the prognostic and predictive value of TP53 aberrations varies substantially by disease entity, molecular context, and the role of this genetic event within the hierarchy of oncogenic drivers in a given tumor. The lack of a clear understanding of whether TP53 mutation represents an initiating or a late acquired event in tumor evolution precludes consideration of TP53-mutated status as a universal adverse prognostic factor across all neoplasms.

Among lymphoproliferative disorders, the most compelling evidence base regarding the clinical significance of TP53 aberrations has been accumulated in chronic lymphocytic leukemia and mantle cell lymphoma. In these entities, TP53 mutations and deletions typically represent a late genetic event acquired during clonal evolution under therapeutic selective pressure, and their detection is associated with primary resistance to standard immunochemotherapy, early relapse, and poor prognosis [4,5]. In clinical practice, assessment of TP53 aberrations is mandatory both at initial diagnosis and at relapse in chronic lymphocytic leukemia and mantle cell lymphoma, and their verification serves as an indication for targeted agents and cellular therapy as alternatives to conventional cytotoxic regimens [20,21].

In aggressive B cell lymphomas, and particularly in DLBCL, the landscape is considerably more complex and nuanced, a reflection of the marked molecular heterogeneity that characterizes this group of tumors [1]. In contrast to chronic lymphocytic leukemia and mantle cell lymphoma, TP53 mutations in DLBCL are frequently detectable at initial diagnosis and may represent either early initiating events or late acquired aberrations. The clinical significance of TP53 mutations in DLBCL is substantially modified by the molecular context of the tumor, including concomitant genetic events, cell of origin, and the presence or absence of biallelic gene inactivation [3,22]. This biological complexity has driven the incorporation into contemporary genomic classifications of a distinct molecular subtype, A53, which encompasses DLBCL cases with TP53 inactivation and associated features of genomic instability, thereby enabling more precise patient stratification and the assembly of biologically homogeneous cohorts for clinical studies [2].

Accumulating evidence has shown that TP53 aberrations in DLBCL are associated not only with adverse prognosis but also with the biology of early treatment resistance [8]. Among patients who fail to respond to first-line therapy, TP53 mutations are identified at the highest frequency and frequently co-occur with alterations in chromatin remodeling processes, including defects in ARID1A and other epigenetic modifiers [23,24]. In this context, our observations contribute to a growing body of evidence supporting the need for biologically guided modifications to first-line therapy in patients with TP53-aberrant DLBCL. Unlike retrospective studies, the prospective application of the LymphGen algorithmic classification enabled the identification of a biologically defined A53 group, characterized not only by TP53 aberrations but also by concomitant features of genomic instability. This approach ensures a more homogeneous molecular population and enhances the biological interpretability of our findings compared with studies based solely on assessment of TP53 mutation status without consideration of the tumor molecular context [9]. Nevertheless, in the absence of randomized control, the observed clinical effect cannot be unequivocally attributed to the addition of decitabine, as it may reflect epigenetic sensitization, the intrinsic sensitivity of the selected subpopulation, or a combination of both.

The CMR rate observed in our cohort is notably higher than the historical outcomes reported for TP53-altered DLBCL treated with standard R-CHOP, a setting in which primary refractory disease and early relapse remain prominent drivers of treatment failure. Consistent with this clinical phenotype, a systematic review and meta-analysis (20 studies; *n* = 2704) reported that TP53-mutated DLBCL has a pooled complete response (CR) rate of 36% (95% CI, 0.32–0.41) and a reduced likelihood of achieving CR (effect estimate 0.39; 95% CI, 0.29–0.53), together with inferior survival (OS HR 2.26, 95% CI 1.90–2.69; PFS HR 1.90, 95% CI 1.49–2.42) compared with TP53–wild-type disease [25]. Beyond single-gene TP53 status, large-scale integrative molecular efforts using the LymphGen framework have clarified that the highest-risk biology is enriched within the A53 genetic class, which is defined largely by TP53 inactivation occurring in the context of extensive copy-number complexity/aneuploidy, i.e., a genomic-instability phenotype. Importantly, the LymphGen classifier is explicitly constrained by the availability of copy-number inputs: because A53 is primarily driven by copy-number features, A53 cannot be predicted when copy-number data are absent (and is excluded from consideration in that scenario), underscoring the methodological importance of centralized, comprehensive molecular profiling when A53-specific risk enrichment is an explicit study goal [26]. The adverse clinical behavior of this genomically unstable subgroup under conventional immunochemotherapy is supported by analyses of uniformly R-CHOP-treated trial cohorts that integrate genetic subtyping with on-treatment PET response assessment. In that study, among all R-CHOP-treated patients (*n* = 592), the C2/A53 subgroup showed significantly inferior outcomes; specifically, LymphGen-A53 vs. the remainder was associated with worse PFS (*p* = 0.004) and OS (*p* = 0.002). Notably, consistent with a high burden of primary refractory disease, only 67% of C2 patients became end-of-treatment PET-negative, compared with 81–88% in other subtypes [27]. Against these historical benchmarks—derived from retrospective and meta-analytic cohorts—our observed 100% CMR in a pilot cohort should be interpreted as hypothesis-generating and requires validation in adequately powered, controlled studies. Nonetheless, the magnitude of the quantitative contrast relative to established TP53-associated and A53-enriched resistance signatures provides a strong rationale to further investigate epigenetic sensitization approaches in this molecularly defined high-risk population, particularly when implemented alongside centralized molecular profiling to ensure precise A53 enrichment and reduce prognostic dilution from biologically heterogeneous TP53-altered cases.

The potential mechanisms of decitabine activity in the setting of TP53 mutations encompass several synergistic biological processes. Inhibition of DNA methyltransferases by decitabine induces reactivation of epigenetically silenced tumor suppressor genes and regulators of apoptosis, thereby partially restoring cell cycle control even in the presence of functional p53 inactivation [17]. This effect is complemented by demethylation of major histocompatibility complex class I molecules on the tumor cell surface, which potentiates their immunological recognition by the T cell system. Critically, induction of the viral mimicry phenomenon also plays a key role: demethylation of endogenous retroviral sequences (ERVs) with subsequent activation of interferon-dependent signaling pathways significantly enhances rituximab-mediated antibody-dependent cellular cytotoxicity and activates innate antitumor immune responses [14,28]. The aggregate of these mechanisms provides a rational biological foundation for combining decitabine with rituximab-containing immunochemotherapy specifically in the patient population with TP53-aberrant DLBCL [15]. At the same time, to confirm the engagement of these mechanisms within the framework of the DAC-R-CHOP protocol, direct translational studies incorporating mandatory biomarker analyses are required, including assessment of global demethylation levels, expression of ERV transcripts, and parameters of interferon responses in tumor tissue and peripheral blood of patients.

Alongside the biological rationale and the emerging data on the therapeutic potential of the DAC-R-CHOP regimen, tolerability is a critical determinant of its practical applicability. The toxicity profile of DAC-R-CHOP in this cohort was as expected and broadly consistent with the characteristics anticipated for anthracycline-containing immunochemotherapy regimens. Hematologic toxicity was universal in nature but was effectively managed through mandatory primary prophylaxis with G-CSF. The addition of decitabine to the R-CHOP backbone resulted in a predictable and clinically meaningful increase in the rate of febrile neutropenia, reaching 100%. This degree of myelosuppression exceeds historical data for standard R-CHOP [29,30] and reflects the expected synergism between the cytotoxic and hypomethylating components. Accordingly, the regimen is associated with a high rate of severe hematologic toxicity that necessitates mandatory primary G-CSF prophylaxis and intensive clinical monitoring. Nevertheless, the complications that arose were effectively managed with broad-spectrum antibacterial therapy and supportive measures. In no case did dose reductions or critical delays in therapy cycles become necessary, and no fatal infectious complications were recorded. The severe gastrointestinal bleeding events observed in 2 patients during induction therapy warrant particular attention. These complications were attributable to local lysis of tumor substrate within the gastric wall, consistent with published data on the risk of hemorrhagic manifestations during effective treatment in patients with baseline gastrointestinal involvement [31,32,33]. Importantly, both episodes were successfully managed in a specialized hospital setting without premature protocol discontinuation, underscoring the manageability of this adverse event under appropriate clinical monitoring.

The results of this proof-of-concept study must be interpreted in light of key limitations. First, the analytic cohort is very small (*n* = 6), resulting in substantial statistical uncertainty around the observed 100% complete metabolic response (CMR) rate (exact Clopper–Pearson 95% CI, approximately 54–100%), limiting generalizability and precluding meaningful assessment of response predictors or infrequent adverse events. Second, the current median follow-up of 6 months is too short to adequately evaluate progression-free survival (PFS), overall survival (OS), or response durability, and relapse events beyond the present observation window cannot be excluded. Third, the single-arm design without a contemporaneous control group precludes confident attribution of the observed outcomes specifically to the addition of decitabine. Fourth, biological heterogeneity within TP53-mutant disease—including variability in mutation location across functional protein domains and differences in allelic status/variant burden—may be particularly consequential in a small sample, in which biologically unique cases can disproportionately influence aggregate estimates [10,34]. Fifth, no dedicated translational biomarker analyses were incorporated to directly interrogate the mechanistic activity of decitabine in this setting. Specifically, we did not evaluate longitudinal changes in DNA methylation, interferon pathway activation, immune-related biomarkers, or circulating tumor DNA (ctDNA) dynamics, which could have provided pharmacodynamic confirmation of on-target epigenetic effects and additional insight into response depth and durability. Notwithstanding these constraints, universal CMR among evaluable patients represents a hypothesis-generating signal in a molecularly defined, high-risk population enriched for TP53 alterations and LymphGen A53 features, in which standard frontline immunochemotherapy has historically been associated with inferior outcomes. Accordingly, these findings should be viewed as supportive of biological plausibility rather than as evidence of durable clinical benefit. Extended follow-up of this cohort is ongoing to better characterize response durability and longer-term outcomes, and prospective validation in larger, adequately powered (ideally controlled) studies is required. Future prospective studies should incorporate predefined longitudinal biospecimen collection to enable integrated pharmacodynamic, methylation, immune-correlative, and ctDNA-based analyses.

Notwithstanding these limitations, our data demonstrate the clinical feasibility and preliminary efficacy of the DAC-R-CHOP regimen in patients with the A53 subtype of DLBCL and provide a rationale for conducting multicenter randomized trials with adequate statistical power and an integrated translational component, aimed at confirming the contribution of epigenetic sensitization and identifying molecular biomarkers of response. More broadly, our findings should be interpreted in the context of an ongoing shift toward molecularly tailored frontline strategies in DLBCL, in which targeted agents are added to standard immunochemotherapy based on genetic subtype rather than cell-of-origin alone. A key proof-of-principle for this approach is provided by the randomized phase II GUIDANCE-01 trial, which implemented a genomics-guided “R-CHOP-X” strategy (including decitabine for TP53-mutant disease) and reported higher complete response rates and improved 2-year survival outcomes relative to R-CHOP alone [35]. Building on these results, a multicenter randomized phase III confirmatory study (GUIDANCE-02) evaluating genotype-guided R-CHOP-X versus R-CHOP is ongoing [36]. In parallel, retrospective genetic subtype analyses of prior randomized trials have also demonstrated that treatment benefit from adding targeted agents to R-CHOP can be subtype-specific (e.g., benefit of ibrutinib in defined genetic subsets in PHOENIX re-analyses), reinforcing the broader rationale for subtype-informed trial design [37]. Complementary integrative work combining genetic subtyping with PET-based response monitoring further supports this evolving paradigm. In a recent Nature Communications analysis integrating LymphGen/DFCI genetic subtypes with interim and end-of-treatment PET across multiple R-CHOP-treated trial cohorts, genetic subtype was shown to add prognostic information and to influence the interpretation and predictive value of PET response, thereby providing a framework for subtype-directed monitoring and subtype-specific clinical trial design, including for genomically unstable high-risk disease [27]. Finally, as emphasized in recent expert perspectives and implementation-focused reviews, broader adoption of molecularly tailored therapy in DLBCL will depend on continued progress toward clinically actionable and consensus genomic classification workflows that can be reliably deployed in real-world practice [2,38].

To address the current lack of mechanistic biomarker data, the planned multicenter phase II study is designed to prospectively incorporate translational correlative analyses with longitudinal biospecimen collection, including serial DNA methylation profiling, interferon pathway activation readouts, immune-related biomarker assessment, and ctDNA-based response and minimal residual disease monitoring. These integrated endpoints should help strengthen biological attribution, support refinement of patient selection, and clarify relationships between molecular response kinetics and the durability of clinical benefit.

Further development of molecularly adapted strategies for A53 variants of DLBCL emerges as a priority direction in overcoming primary resistance to immunochemotherapy and improving long-term outcomes in this patient population.

## 5. Conclusions

Molecular genetic stratification of DLBCL using the LymphGen classification identifies the A53 subtype, which is associated with an extremely poor prognosis under standard therapy. In this prospective pilot study, the molecularly adapted DAC R CHOP regimen demonstrated marked antitumor activity, with complete metabolic response achieved in all patients and a clinically manageable toxicity profile. Despite the limitations imposed by the small sample size, absence of a control group, and short follow up, these findings support further clinical evaluation of this approach in larger prospective multicenter trials incorporating translational biomarkers of epigenetic sensitization.

## Figures and Tables

**Table 1:** *Characteristics of Patients with DLBCL and TP53 Gene Mutation.*

Patient	IPI	Disease Stage	ECOG	Extranodal Involvement	Exon	cDNA	Protein	Allelic Burden	Clinical Significance	Response to DAC-R-CHOP	Status
1	high	IV	3	Yes	4	c.515T > A	p.Val172Asp	57%	pathogenic	CMR	Alive
2	low	II	2	No	4	c.380C > A	p.Ser127Tyr	40%	pathogenic	CMR	Alive
3	high	IV	4	Yes	6	c.749C > T	p.Pro250Leu	27%	pathogenic	CMR	Alive
4	high	IV	3	Yes	7	c.742C > T	p.Arg248Trp	5%	pathogenic	CMR	Alive
5	low	II	1	No	4	c.488A > G	p.Tyr163Cys	20%	pathogenic	CMR	Alive
6	high	IV	3	Yes	7	c.742C > T	p.Arg248Trp	11%	pathogenic	CMR	Alive

**Table 2:** ***Toxicity Profile of Therapy in the Study Cohort.***

Adverse Event	Grade III–IV, *n* (%)
Anemia	6 (100)
Neutropenia	6 (100)
Thrombocytopenia	6 (100)
Febrile neutropenia	6 (100)
Gastrointestinal bleeding	2 (33)

## Data Availability

The data presented in this study are available on reasonable request from the corresponding author. The data are not publicly available due to ethical and privacy restrictions.

## Abbreviations

CMR	complete metabolic response
CTCAE	Common Terminology Criteria for Adverse Events
DAC-R-CHOP	decitabine plus R-CHOP
DLBCL	diffuse large B-cell lymphoma
ECOG	Eastern Cooperative Oncology Group
ERV	endogenous retrovirus
FFPE	formalin-fixed, paraffin-embedded
G-CSF	granulocyte colony-stimulating factor
IPI	International Prognostic Index
IQR	interquartile range
NGS	next-generation sequencing
PET/CT	positron emission tomography/computed tomography
R-CHOP	rituximab, cyclophosphamide, doxorubicin, vincristine, prednisone
VAF	variant allele frequency
WHO	World Health Organization
